# Clarification for Late Trial Registration and Additional Study Limitations

**DOI:** 10.1001/jamanetworkopen.2025.16123

**Published:** 2025-05-06

In the Original Investigation titled “Probiotics and Fever Duration in Children With Upper Respiratory Tract Infections: A Randomized Clinical Trial,”^1^ published on March 14, 2025, the authors have provided clarifications to explain why this trial registered after the trial was completed and to address additional study limitations. The authors have explained these clarifications in a Comment posted with the article.^2^
